# Analysis of HIV Correlated Factors in Chinese and Vietnamese Female Sex Workers in Hekou, Yunnan Province, a Chinese Border Region

**DOI:** 10.1371/journal.pone.0129430

**Published:** 2015-06-08

**Authors:** Junjie Wang, Guowei Ding, Zhibin Zhu, Chunlian Zhou, Ning Wang

**Affiliations:** 1 National Center for AIDS/STD Control and Prevention, Chinese Center for Disease Control and Prevention, Changping District, Beijing, P.R. China; 2 Hekou Center for Disease Control and Prevention, Hekou, Yunnan, P.R. China; 3 Department of Nosocomial Infection Prevention and Control, Beijing Friendship Hospital, Capital Medical University, Beijing, P.R. China; Rollins School of Public Health, Emory University, UNITED STATES

## Abstract

**Objectives:**

To assess the prevalence and correlated factors of HIV-1 among Chinese and Vietnamese female sex workers (FSW) in the border county of Hekou, Yunnan province, China.

**Methods:**

A cross-sectional survey was conducted collecting information on demographics, sexual behavior, medical history, and drug use. Blood samples were obtained to test for HIV/STIs. Multivariate logistic regression model was used to examine associations between factors and HIV-1 infection.

**Results:**

Of 345 FSWs who participated in this study, 112 (32.5%) were Chinese and 233 (67.5) were Vietnamese. Vietnamese FSWs were significantly more likely to be HIV-1 positive (7.7%) compared with Chinese FSWs (0.9%) (p = 0.009). In multivariate analysis, sexual debut at age≤16 (OR 3.8: 95% CI: 1.4, 10.6), last client’s payment <150 RMB ($22 USD) (OR: 5.2, 95% CI; 1.7, 16.6), and HSV-2 (OR: 12.3; 95% CI: 1.6, 94.8) were significant for HIV-1 infection.

**Conclusions:**

Differences in HIV prevalence in Vietnamese and Chinese FSWs may be indicative of differential risk. It is important to characterize the nature of trans-border transmission in order to gain a better understanding of the potential impact on the international HIV epidemic. Understanding the correlated factors for HIV in Vietnamese and Chinese FSWs is important for designing interventions for this vulnerable population.

## Introduction

It is recognised that cross-border sex workers have high rates of both HIV/STD prevalence and incidence, and that they play an important role in transmitting HIV across national borders.

Thomas L. et al[1]. concluded that HIV prevalence was as high as 6% among female sex workers (FSWs) in two large cities on the US-Mexico border. Another survey[2] in the U.S.-Mexico border region from 2003–2006 concluded that 80% of 4,279 cases of HIV infection were transmitted through sexual contacts, including MSM and high-risk heterosexual contacts. In a molecular-epidemiological survey[3], seven HIV-1 hypotypes were detected in the border areas of Yunnan, China, which may have been spread to Yunnan from the neighboring countries. A study conducted in the five border provinces of Vietnam concluded the prevalence of HIV among FSWs was 4.5% in these areas[4]. The factors of high prevalence in border areas are multiple, such as drug use, commercial sex and migration, thus, intensifying the potential risk of HIV transmission in these regions[5–11]. A systematic review of the studies conducted on the Indo- Nepal Border concluded that high prevalence of HIV was associated with male labour migrants and commercial sex at the border area[12].

With the development of commercial relationships between China and Vietnam, there are more and more people moving across the border of the two countries, including mobile female sex workers. Yunnan province shares a 710 kilometer land border with Vietnam, and is located near the “Golden Triangle” of Myanmar, Thailand and China’s border[13]. Yunnan has the highest HIV/AIDS prevalence rates in China. Although many cases in the region were initially concentrated among injection drug users (IDUs), sexual transmission has been playing an increasingly greater role since 2005[14]. Hekou in Yunnan province is an important border-trade and tourist county on the Chinese-Vietnamese border with a population of 103,400, which has an estimated mobile population of 28,661[15].The land border extends for 193 kilometers between Hekou County and Lao Cai Province in Vietnam[16].

It has been reported that there is an underground sex industry in Hekou dominated by Vietnamese female migrants who have been serving locally as sex workers ever since Hekou was opened to the outside world in 1992[16]. It has been estimated that there are over 1,000 FSWs in Hekou, including both 500 Vietnamese and 500 Chinese approximately[16]. Most Vietnamese FSWs come from northern provinces in Vietnam such as Lao Cai, Ha Giang, Lai Chau, and Yen Bai provinces. Most of the Vietnamese FSWs gather in a border trade market named “Vietnamese Street” for work and living[16]; in contrast, their Chinese colleagues often scatter all over the county. Little is known about the HIV/AIDS infection correlated factors of FSWs based on the Vietnam-China border in Hekou. Vietnamese FSWs residing in Hekou may experience different risks than their Chinese counterparts. In addition, the specific risk factors for HIV and sexually transmitted infection (STI) acquisition in this population are, as of yet, unknown. This study focuses on the HIV/AIDS prevalence of FSWs and attempts to identify the factors which impact the HIV/AIDS transmission in this border area.

## Methods

This cross-sectional study of FSWs in Hekou was conducted in May and June of 2009. Study participants were recruited by local outreach workers and health officials. Women were considered eligible for study participation if they were at least 16 years of age, provided sexual services in exchange for money in the past 6 months, and were Chinese or Vietnamese. Convenience sampling was used in recruiting. All those eligible for study participation were encouraged to take part in the interview. Before being interviewed or having specimens taken, participants provided written voluntary informed consent. Data were collected on demographics, sexual behavior, medical history, and drug use through face-to-face interviews conducted by study staff who were fluent in both Chinese and Vietnamese. Chinese participants were interviewed in Mandarin, and Vietnamese participants were interviewed in Vietnamese. All study staff were trained in standardized methods of data collection.

Participants were compensated 50 RMB ($7 USD) for participation in this study. Participants were scheduled for a follow-up visit four to six weeks from the date of the study visit for post- test counseling. This study received ethics approval from both the China CDC and Yunnan Province CDC institutional review boards. By Chinese law, participants who were between 16–18 years were considered as having full capacity for civil conduct if they relied on their own labor income, and therefore, they signed the same written voluntary informed consent as participants older than age 18. his also was approved by the China CDC and Yunnan Province CDC institutional review boards.

All laboratory specimens were collected and refrigerated immediately by trained physicians. The following specimens were collected: 7 ml of blood were collected by venipuncture for diagnosis of HIV-1, HSV-2 and syphilis; 10–15 ml of urine were collected for opiate screening; Vaginal swab samples were collected for diagnosis of Trichomonas vaginalis, bacterial vaginosis and yeast infections. The Hekou County CDC laboratory performed HIV-1 ELISAs, HSV-2 ELISAs, RPRs, wet mounts for Trichomonas vaginalis, bacterial vaginosis, yeast infections, and urine drug tests. HIV Western blot and TPPA for syphilis were tested at the Honghe Prefecture CDC laboratory. Blood and vaginal specimens were sent from the study site to the Hekou County CDC laboratory by car every four hours and were stored in the Hekou County CDC laboratory at -20°C. Blood and vaginal specimens were transported to the Honghe Prefecture CDC laboratory every week by car. The appropriate temperature was maintained during the transport of all specimens.

Plasma specimens were screened for HIV-1 antibodies by enzyme-linked immunosorbent assay (ELISA: Organon Teknika, Boxtel, Co., Ltd., the Netherlands), and positive tests were confirmed by HIV-1/2 Western blot assay (HIV Blot 2.2 WBH; Genelabs Diagnostics, Singapore). Plasma specimens were also tested for antibodies to herpes simplex virus 2 (HSV-2) by ELISA (Herpe Select-2 ELISA IgG; Focus Technologies. Cypress, CA). Syphilis was tested using rapid plasma reagin (RPR; RPR Diagnostics kit; Xinjiang Xindi, China) for Treponema pallidum and positive specimens were confirmed using T. pallidum particle assay (Serodia; Fujirebio, Inc., Fuji, Japan). Participants with plasma positive for both T. pallidum particle assay and RPR were considered positive for syphilis. Samples were considered Trichomonas vaginalis (TV) positive if motile organisms in wet mounts were seen under 100 times magnified microscope. Bacterial vaginosis (BV) was diagnosed using Amsel’s criteria[17]. Yeast infections were diagnosed using microscopy with a potassium hydroxide (KOH) solution to examine for the presence of pseudohyphae and budding yeast cells. Cervicitis and genital warts were diagnosed by visual inspection. Urine specimens were screened for opiates by morphine gold conjugate test strip method (Acon MOP). Participants were considered illegal drugs users if they self-reported illegal drugs use or tested urine-positive by morphine gold conjugate test strip method; injection drug use status was based on self-report.

Data from the questionnaires and laboratory tests were entered into EpiData 3.1 (Odense, Denmark). All data were analyzed using SAS 9.1 (Cary, NC). Chi-squared and Fisher’s exact tests (FET) were used to examine associations in univariate analyses. Wilcoxon non-parametric tests were used to examine the association for continuous non-normal data. Multivariate logistic regression model was used to examine associations between factors and HIV-1 infection. Whether or not infected with HIV-1 was considered as the dependent variable in the established regression model, with others variables being as independent variables. Variables significant (p<0.2) in univariate analyses were considered for inclusion in the multivariate regression model. Variables were entered and eliminated from the model in a stepwise manner with entry and exit criteria of p<0.2.

## Results

There were three geographically concentrated, market-style commercial sex venues in downtown Hekou, where FSWs live and work. Most Vietnamese FSWs worked in one of these three sex markets. However, most of the Chinese FSWs worked in night clubs, karaoke halls, hotels, hair and beauty salons and temporary sublets. Local outreach workers and health officials informed the bosses of all the downtown sites, which were believed to have FSWs, including the 3 sex markets, 4 night clubs, 21 hotels, 16 karaoke halls,18 hair and beauty salons, 5 temporary sublets, and 2 public parks, to inform their FSWs about the study.

A total of 353 FSWs were approached to participate in this study. Six FSWs who were younger than 16 years of age were excluded. There were 347 FSWs who provided informed consent and finished the questionnaire interview. One FSW’s blood sample was not collected because she fainted during blood sample collection, and three FSWs’ vaginal swab samples were not collected during the first enrollment because of menstruation. After fifteen days, the three FSWs were approached again, but one could not be found and only two had vaginal swab samples were collected. There were a total of 345 FSWs who finished the questionnaire interview and provided biological specimens (Data set used for the analyses of Chinese and Vietnamese FSWs was in S1 Appendix). Of the 345 FSWs, 247 were from the three sex markets, 54 from night clubs and karaoke halls, 15 from hotels, 23 from hair and beauty salons, 5 from temporary sublets, and 1 worked in public parks.

Of the 345 study participants, 112 (32.5%) were Chinese and 233 (67.5%) were Vietnamese. Differences in characteristics of Chinese and Vietnamese FSWs are shown in Table 1. Age, education, marital status, residence, and alcohol use were different between Chinese and Vietnamese FSWs. Vietnamese FSWs were younger and acquired less education than their Chinese counterparts. More Vietnamese were not married/cohabitating and resided in the commercial sex market. The Chinese FSWs had a higher percentage of alcohol use. There was no difference in illicit drug use.

**Table 1:** Demographic characteristics in FSWs of Chinese (N = 112) and Vietnamese (N = 233).

Variable	Status	Chinese no.(%)	Vietnamese no.(%)	P value
**Median age (range)**	-	22 (17–46)	20 (16–42)	<0.0001
**Age**	<21years	32 (28.6)	124 (53.2)	<0.0001
**Ethnicity**	Ethnic majority*	73 (65.2)	155 (66.5)	0.80
**Education**	≤6 years	21 (18.8)	78 (33.5)	0.005
**Marital status**	Married or cohabitating	23 (20.5)	24 (10.3)	0.01
**Residence**	Commercial sex market	20 (17.9)	227 (97.4)	<0.0001
**Alcohol use**	Yes	93 (83.0)	38 (16.3)	<0.0001
**History of illicit drug use**	Yes	2 (1.8)	2 (0.9)	0.45

*Han Chinese or Kinh Vietnamese.

Table 2 illustrates the sexual behaviors of Chinese and Vietnamese FSWs. Age of sexual debut, duration of sex work, condom use with last client, convenient access to condoms at work, ever had a steady sexual partner, currently have a steady sexual partner, history of pregnancy, number of clients in the last day, amount of last client’s payment, sexual acts with the last client, and number of douches per day were different between Chinese and Vietnamese FSWs.

**Table 2:** Sexual risk behaviors in FSWs of Chinese (N = 112) and Vietnamese (N = 233).

Variable	Status	Chinese no.(%)	Vietnamese no.(%)	P value
**Age of sexual debut**	≤16 years old	16 (14.3)	58 (24.9)	
	>16 years old	96 (85.7)	175 (75.1)	0.02
**Mean/Median month of sex work**	-	9.9	2.3	<0.0001
**Duration of sex work**	≤3months	29 (25.9)	119 (51.1)	
	>3months	83 (74.1)	114 (48.9)	<0.0001
**Main contraception method**	Condom	107 (95.5)	222 (95.3)	
	Other	5 (4.5)	11 (4.7)	0.92
**Condom use with the last client**	Yes	112 (100.0)	210 (90.1)	
	No	0 (0.0)	23 (9.9)	0.0006
**Convenient access to condoms at work**	Yes	65 (58.0)	228 (97.9)	
	No	47 (42.0)	5 (2.2)	<0.0001
**Ever had a steady sexual partner**	Yes	105 (93.8)	154 (66.1)	
	No	7 (6.3)	79 (33.9)	<0.0001
**Currently has a steady sexual partner**	Yes	64 (57.1)	96 (41.2)	
	No	48 (42.9)	137 (58.8)	0.005
**History of pregnancy**	Yes	68 (60.7)	92 (39.5)	
	No	44 (39.3)	141 (60.5)	0.0002
**Had at least 5 clients in the last day**	Yes	5 (4.5)	181 (77.7)	
	No	107 (95.5)	52 (22.3)	<0.0001
**Last client’s payment**	≤150 RMB ($22 USD)	14 (12.5)	122 (52.4)	
	≥150 RMB ($22 USD)	98 (87.5)	111 (47.6)	<0.0001
**Sexual acts with the last client**	Oral and vaginal sex	3 (2.7)	107 (45.9)	
	Only vaginal sex	109 (97.3)	126 (54.1)	<0.0001
**Repeat customer**	≥1	51 (45.5)	103 (44.2)	
	0	61 (54.5)	130 (55.8)	0.82
**Douches more than once a day**	Yes	20 (17.9)	205(88.0)	
	No	92 (82.1)	28(12.0)	<0.0001

Vietnamese FSWs had higher overall morbidity compared with Chinese FSWs as shown in Table 3. Vietnamese FSWs had a higher prevalence of HIV-1, BV, and yeast infections. However, they had a lower prevalence of TV.

**Table 3:** HIV-1 and other genital diseases in FSWs of Chinese (N = 112) and Vietnamese (N = 233).

Variable	Status	Chineseno.(%)	Vietnamese no.(%)	*P* value
**HIV-1**	Yes	1 (0.9)	18 (7.7)	0.009
**HSV-2**	Yes	59(52.7)	142(60.9)	0.14
**Syphilis**	Yes	1 (0.9)	1 (0.4)	0.54*
**Trichomonas**	Yes	5 (4.5)	2 (0.9)	0.07*
**Genital warts**	Yes	2 (1.8)	7 (3.0)	0.76*
**Cervicitis**	Yes	7 (6.3)	46 (19.7)	0.001
**Bacterial vaginosis**	Yes	1 (0.9)	13 (5.6)	0.08*
**Yeast infection**	Yes	10 (8.9)	38 (16.3)	0.06

*p-value calculated using Fisher’s exact test.

Table 4 shows the correlated factors for HIV-1 infection. In univariate analysis, correlated factors for HIV-1 infection were being a Vietnamese FSW, residing in the commercial sex market, having a history of illicit drug use, reporting a sexual debut at age 16 or younger, douching more than once a day, having more than 5 clients in the last day, last client’s payment amount less than 150 RMB ($22 USD), and having HSV-2. Condom use with the last client was found to be protective of HIV-1 infection. In the multivariate model sexual debut at age 16 or younger, last client’s payment less than 150 RMB ($22 USD), and HSV-2 infection were significant for HIV-1 infection.

**Table 4:** Correlated factors for HIV-1 infection in overall FSWs.

Variable	OR (95% CI)	AOR (95%CI)
**Country of origin (Vietnam vs. China)**	9.3 (1.2, 70.5)§	
**Age (<21years vs. ≥21 years)**	0.5 (0.2, 1.5)	
**Ethnicity (majority◇ vs. minority)**	1.5 (0.5, 4.2)	
**Education (≤6 years vs. >6 years)**	1.5 (0.6, 3.9)	
**Married or cohabitating**	1.8 (0.6, 5.5)	
**Resides at commercial sex market**	7.6 (1.0, 57.9)‡	
**Alcohol use**	0.4 (0.1, 1.3)	
**History of illicit drug use**	6.0 (0.6, 60.4)†	
**Condom used as a main contraception method**	0.4 (0.08, 1.8)	
**Duration of sex work (≤3months vs. >3 months)**	0.5 (0.2, 1.3)	
**Age of sexual debut (≤16 years old vs. >16 years old)**	4.5 (1.8, 11.7) §	3.8 (1.4, 10.6) §
**Has ever had a steady sexual partner**	1.8 (0.5, 6.4)	
**Currently has a steady partner**	0.8 (0.3, 2.1)	
**Douches more than once a day**	4.8 (1.1, 21.2) ‡	
**At least 5 clients in the last day**	3.4 (1.1, 10.5) ‡	
**Last client’s payment less than 150 RMB ($22 USD)**	6.4 (2.1, 19.6) §	5.2 (1.7, 16.6) §
**Condom use with the last client**	0.2 (0.07, 0.8) ‡	
**Convenient access to condoms at work**	3.3 (0.4, 25.6)	
**Has at least one repeat customer**	1.1 (0.4, 2.8)	
**HSV-2**	14.1 (1.9, 106.6) §	12.3 (1.6, 94.8) ‡
**Syphilis**	3.3 (0.2, 71.7)*	
**Trichomonas vaginitis**	1.1 (0.06, 19.8)*	
**Bacterial vaginosis**	1.3 (0.2, 10.8)	

* 0.5 used to calculate OR in cells with a count of 0.

† p-value <0.2.

‡ p-value <0.05.

§ p-value <0.01.

◇Han Chinese or Kinh Vietnamese.

## Discussion

In this study, numerous differences were found in demographics, sexual behaviors, and HIV-1 prevalence between Vietnamese and Chinese FSWs in Hekou. Vietnamese FSWs had a much higher HIV-1 prevalence compared with their Chinese counterparts (7.7% vs. 0.9%). Significantly, the HIV prevalence in Vietnamese FSWs residing in Hekou is higher than that reported for FSWs in all of Vietnam and China[18, 19]. Previous research has found slightly higher HIV prevalence rates among FSWs in other cities in Yunnan province, however, unlike the current study’s population, that reported low drug use, these other studies found that illegal drug use was the primary risk factor for HIV infection[20, 21].

The HIV prevalence found among Chinese FSWs in this study was higher than the national estimate of L Zhang, et al[22], but lower than that reported by Baral S, et al[23]. Wang L, et al[24] reported HIV prevalence of FSWs in China decreased from 0.6% in 2008 to 0.3% in 2012. Li D, et al[25] reported HIV prevalence among street-based FSWs in China changed from 1.5% in 2010 to 1.4% in 2011 and 2.3% in 2012. Sample selection may be an important factor affecting the estimated HIV prevalence of Chinese FSWs in the current study. If more street-based FSWs were recruited, maybe more HIV positive cases would have been found in this study. Moreover, FSWs were mostly recruited from the urban area not the rural area of Hekou and the HIV prevalence may have varied between these areas. HIV prevalence in China varies geographically, and Yunnan has a particularly high HIV prevalence, which has its origins among people who inject drugs, but Yunnan also has higher HIV prevalence among FSWs compared to other regions[22].

Other studies have found that sex trafficking is a common risk factor for HIV infection[26–28] and trafficked women have been shown to be at greater risk for HIV and other STIs compared with those who enter commercial sex voluntarily[29]. Although their mode of immigration into China was not explored during this study, there is a possibility that these Vietnamese women were trafficked across the border[16]. During the process of forced or misinformed migration and the life that follows, trafficked women are more likely to be exposed to a number of traumatic experiences which may include but is not limited to violence, sexual abuse, rape, or forced loss of virginity[30]. Those who have been abused or have experienced traumatic events have been found to be at increased risk for HIV acquisition[31].

Therefore, further research is needed to explore the reasons for and modes of immigration to Hekou by Vietnamese FSWs, as the difference in HIV prevalence between Vietnamese FSWs and Chinese FSWs may be indicative of differential risk in the client base.

The main predictors of HIV infection for all FSWs were sexual debut at age 16 or younger, last client’s payment less than 150 RMB, and HSV-2 infection. Other studies have also found that various factors put those with younger age of sexual debut are at increased risk for HIV infection[32–34]. Previous research has also found that those who had sex at an earlier age are less likely to use condoms[33, 35, 36]. A study in South Africa determined that women who had an earlier sexual debut were more likely to have been forced to have sex and were therefore less likely to negotiate condom use[33]. South African studies have found that younger women oftentimes do not negotiate condom use in sexual relationships because they are afraid of violent repercussions from their sexual partners[37, 38]. Moreover, younger women may also have less knowledge about safe sexual practices and lower self-efficacy compared with older women[39, 40]. Further research that delves into the dynamics of sexual abuse, interpersonal violence, and condom use of Hekou FSWs is needed.

Low payment has been found to be a risk factor for HIV infection[4, 41–43], but this finding is not consistent[44]. This may be because those clients who cannot afford to pay more than 150 RMB may be poorer, have lower education, and be at greater risk than those who can afford to pay more. It has been indicated by some studies that HIV infecting was associated with acquiring HSV-2. Genital ulcers caused by HSV-2 may lead to the easier acquisition of HIV[45–47].

This study provides new insights into HIV-related risk behaviors of FSWs working in a border region of China and Vietnam. However, this study is subject to several limitations. First, the marginalized and highly mobile nature of the FSW population in China restricted our ability to sample a truly random population. It is possible that FSW included in our sample were systematically lower risk FSW who were therefore more accessible and willing to participate in our research. Second, as with all research concerning taboo or sensitive behaviors, social desirability may have resulted in under reporting of risky behaviors. Although survey questions were designed to collect information on more recent events to minimize recall bias, validity of this information is difficult to verify. Finally, the relatively low number of outcomes (19/324 participants were HIV-1 antibody positive) may have caused potential confounding in the multiple logistic regression model. The interaction between factors and lower statistical power due to the relatively small sample size may have led to some variables, such as country of origin, residence, douching behavior, number of clients, condom use which were significant (p<0.2) in univariate analyses, to be eliminated from the model. Moreover, the cross-sectional study cannot confirm the causal relationship of factors and only provides information on correlations.

Understanding the correlated factors for HIV in Vietnamese and Chinese FSWs is important for designing interventions for this vulnerable population. It is also important to characterize the nature of trans-border transmission in order to gain a better understanding of the potential impact on the international HIV epidemic.

## Supporting Information

S1 Appendix Data set used for the analyses of Chinese and Vietnamese FSWs. (XLS)
